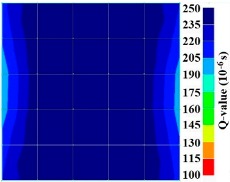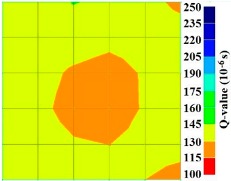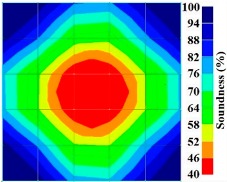# Development of a Tomography Technique for Assessment of the Material Condition of Concrete Using Optimized Elastic Wave Parameters

**DOI:** 10.3390/ma9040291

**Published:** 2016-04-15

**Authors:** Hwa Kian Chai, Kit Fook Liu, Arash Behnia, Kobayashi Yoshikazu, Tomoki Shiotani

**Affiliations:** 1 Department of Civil Engineering, University of Malaya, Kuala Lumpur 50603, Malaysia; chris.liukf@gmail.com; 2 Discipline of Civil Engineering, School of Engineering, Monash University Malaysia, Selangor 46150, Malaysia; 3 Department of Civil Engineering, Nihon University, Tokyo 101-8308, Japan; kobayashi.yoshikazu@nihon-u.ac.jp; 4 Department of Civil and Earth Resources Engineering, Kyoto University, Kyoto 615-8530, Japan; shiotani.tomoki.2v@kyoto-u.ac.jp

**Keywords:** tomography, honeycomb, pre-stressed concrete (PC), imaging algorithm, ray tracing, wave propagation

## Abstract

Concrete is the most ubiquitous construction material. Apart from the fresh and early age properties of concrete material, its condition during the structure life span affects the overall structural performance. Therefore, development of techniques such as non-destructive testing which enable the investigation of the material condition, are in great demand. Tomography technique has become an increasingly popular non-destructive evaluation technique for civil engineers to assess the condition of concrete structures. In the present study, this technique is investigated by developing reconstruction procedures utilizing different parameters of elastic waves, namely the travel time, wave amplitude, wave frequency, and Q-value. In the development of algorithms, a ray tracing feature was adopted to take into account the actual non-linear propagation of elastic waves in concrete containing defects. Numerical simulation accompanied by experimental verifications of wave motion were conducted to obtain wave propagation profiles in concrete containing honeycomb as a defect and in assessing the tendon duct filling of pre-stressed concrete (PC) elements. The detection of defects by the developed tomography reconstruction procedures was evaluated and discussed.

## 1. Introduction

There are various NDT techniques such as impact echo (IE), pulse eddy-current (PEC), and acoustic emission (AE), that can be used either locally or globally [1,2,3,4,5,6,7,8]. The elastic wave tomography (EWT) technique is an emerging local non-destructive testing (NDT) technique for civil engineering applications which utilizes the principal of elastic wave propagation to detect defects in a medium. Each of the above mentioned techniques can be used for specific conditions. For example, IE can be used to find out the thickness of different layers of medium, or PEC can be utilized for surface crack detection, whereas the EWT technique uniquely facilitates visualization inspection so that anomalous regions or distribution of physical properties in the measured object can be visualized [9]. Therefore, it can be utilized to evaluate the soundness of a concrete medium by analyzing the changes in stress wave propagation.

To plot a tomogram there are several techniques. For example, by using pulse eddy-current technique an induction can be made to image electromagnetic properties [6]. This technique makes use of magnetic sensors which bring many advantages but there are some difficulties in image resolution improvement. On other hand, EWT can be performed by piezoelectric sensors which utilize elastic wave properties to plot a tomogram.

Tomography utilizing elastic wave propagation has shown a promising trend in determining the size and location of defects in concrete. Defects such as crack, inclusions, air voids and other objects located inside a concrete medium may cause local changes to the medium density, physico-mechanical properties, and acoustic properties, causing changes in stress wave property distribution throughout the medium [10,11]. Application of EWT technique for detecting defect in concrete structures has been investigated, e.g., Aggelis and Shiotani adopted the technique to successfully visualize a crack in concrete [12]. Furthermore, the efficacy of remedial action on the detected crack was shown by EWT visualization analysis by the same researchers. On the other hand, Momoki *et al.* implemented the EWT technique for soundness evaluation of the concrete piers of a water intake facility by carrying out a series of large-scale measurements [13].

Up to date, as far as the EWT technique is concerned, the velocity distribution within the medium is reconstructed by using a numerical based identification technique. One of the common identification techniques is the Simultaneous Iterative Reconstruction Technique (SIRT). The techniques iteratively update the velocity distribution to minimize the difference between theoretical and observed travel time, for obtaining the optimum wave velocity distribution. To obtain the theoretical velocity, the ray trace technique is employed to acquire the travel time and distance of the wave [9,14]. Apart from those studies dedicated to the development of identification techniques [9], there are other imperative concerns which have been investigated. For instance, the excitation source characteristic showed a significant influence on the waves’ velocity distribution tomogram. Aggelis, in his study, concluded that a higher frequency trigger source produces higher quality tomograms and the element size of the tomogram should be less than the receiver separation distance to overcome the reduced capacity of lower frequency [15]. In addition, the nature of the material is another concern, particularly when mediums with lower material homogeneity material such as concrete are being investigated. Therefore, it was suggested by Kim and Fratta [16] that the ratio between the inclusion size to wavelength of the wave source should be near to 1(d/λ ≈ 1). This is because long wavelengths could not yield appropriate visualization due to the diffraction regime. Moreover, other influential parameters that may affect the accuracy of tomography such as the adequateness of ray path coverage, sensor placement, and number of reading data have been investigated [17].

However, to the best of the authors’ knowledge, the existing literature has investigated the elastic time tomography for wave velocity distribution, while the potential of other parameters of elastic waves have been overlooked. Therefore, this study with two main objectives was conducted. The first was to investigate the applicability of the other elastic wave parameters such as amplitude, frequency and medium quality factor (Q-value) to provide visualized tomograms other than wave velocity. The second objective was to conduct a practical methodology for 2D tomography analysis which can be practiced in large scale structures where accessibility to the entire structures might not be possible to perform a 3D tomography analysis.

## 2. Hypothesis and Proposed Algorithm

**Figure 1:**
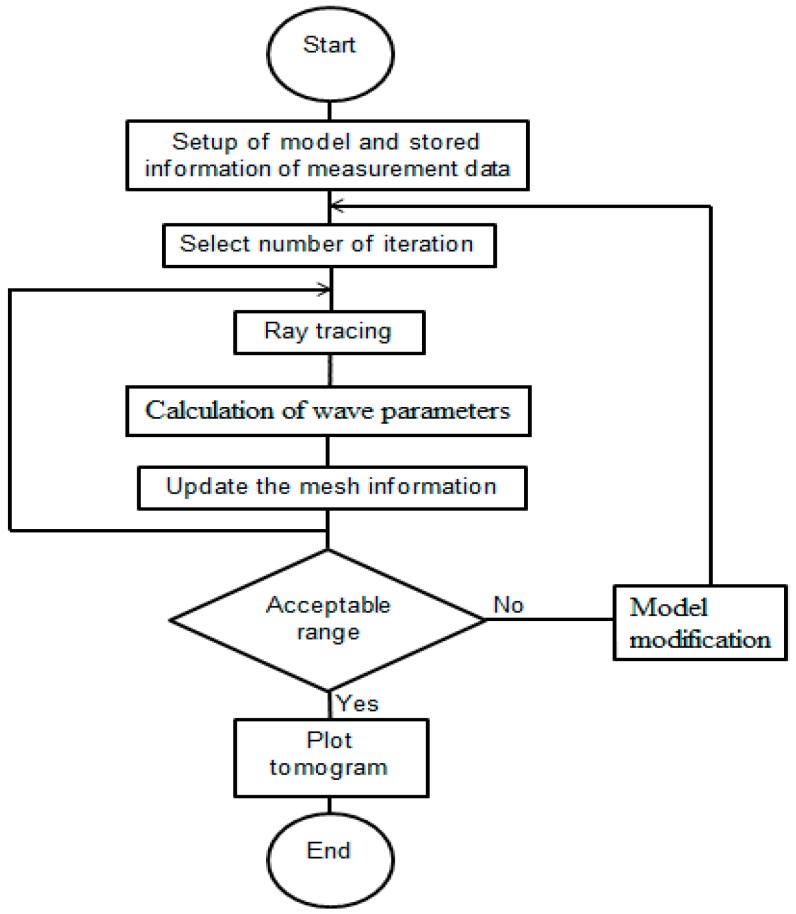
Reconstruction of elastic wave tomography algorithm.

In this study, a computer algorithm was developed to process large amount of elastic wave data for the purpose of tomography reconstruction. Figure 1 shows the standard procedures. The aim was to plot each element of a discretized cell of measurement with the specific elastic wave parameters (e.g., velocity) and to see which magnitude would change according to the local variation of the concrete acoustic properties.

**Figure 2:**
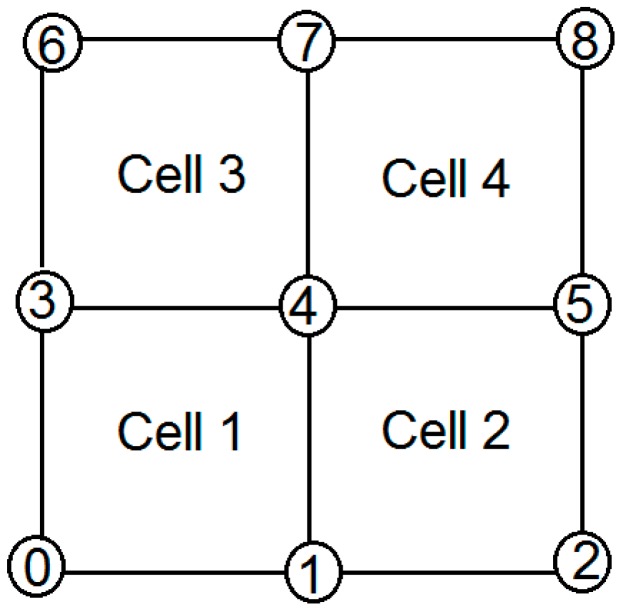
Illustration of assigned number of nodes and number of cells.

To plot a precise tomogram for each element of a discretized cell, it is necessary to assign nodes and define the trigger and receiving points in the model representing the measured area. This is exemplified by a four-element area with nine nodes as in Figure 2, in which each element is assigned a value, e.g., velocity that represents the condition of the measured area. A tomogram is plotted based on the value of each element, indicating the soundness of the measured medium. The developments of algorithms are based on the ray-tracing approach and the simultaneous iterative reconstruction technique (SIRT). The algorithm required information such as the dimension of an object and the mesh dimension in order to distribute node and cell numbers. However, with increasing refinement of the mesh, the accuracy of the tomography can be improved.

Beside velocity, the amplitude and frequency of the elastic waves are the other two parameters examined for tomography reconstruction. The essence of this approach originated from wave energy diffraction encountering abnormality within the propagated medium [18]. Therefore, these two parameters are presented in the form of an attenuation coefficient, which is related to the amount of energy being scattered and diffracted in the measured area.

### 2.1. Ray-Tracing and Mathematical Theory of Elastic Wave Method

#### 2.1.1. Fundamental Concepts and Hypothesis

Ray tracing turns out to be a powerful technique for predicting the possible travel path of elastic waves in a medium so that accurate information about any change in the wave propagation behavior can be accurately quantified. The technique relies on analysis of various mechanical and elastic properties of regions and interfaces the wave is passing through to construct a ray-path [19]. In a conventional ray-tracing algorithm, the wave velocity distribution is reconstructed by minimizing the differences in the observed and theoretical travel times. Therefore, based on this principle, the proposed algorithm in this study determines and updates the wave propagation path in an iterative computational method. This allows identification of the wave travel path and its length, which is often found to be in a non-linear trend due to the inhomogeneity introduced by defect or anomaly in a medium. With the incorporation of the ray-tracing principle, the developed algorithm is capable of determining the interceptions between element boundaries for each possible ray path.

**Figure 3:**
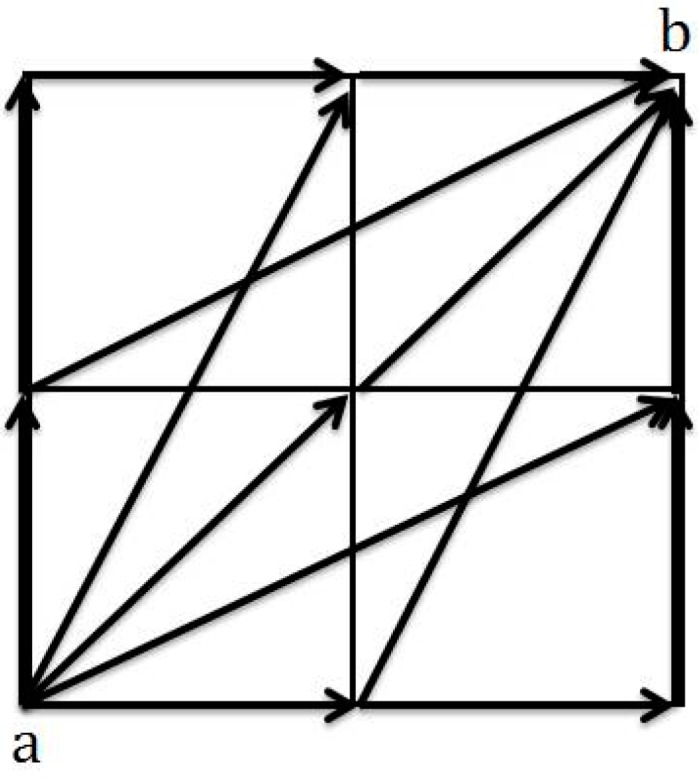
Illustration of several prospective travel paths of waves that propagate from node “a” to “b”.

To describe ray-tracing, an example of a four-cell medium is shown in Figure 3. At least seven prospective travel paths for wave propagation from node “a” to node “b” can be identified. The path that gives the shortest travel time is selected as the designated travel path for wave propagation. After travel path selection, the tomography reconstruction process is initiated. This is a process whereby the information of all elements in a meshed model is updated by minimizing the difference between observed and theoretical values [12]. The process continues in an iterative manner in order to obtain the best possible wave path that could provide accurate information for tomography reconstruction within the acceptable error range. The visualization of tomography can be improved if wave information covers the whole testing area [20,21].

#### 2.1.2. Elastic Wave Tomography Principle

To plot a travel time tomogram, the travel time of a wave along a path can be taken as input data and the inverse of wave velocity, which is known as slowness, is used as the estimated characteristic of the measured object. Therefore, to deal with the inverse problem ray-tracing is utilized. For SIRT-based ray tracing, Hyugen’s principle is employed to justify the propagation of the wave in a two dimensional space which is partitioned into square cells. The theoretical travel time (T_ci_) from the emission source to the receiver can be calculated as follow.

(1) $$T_{\mathrm{ci}}=\sum^{\text{​}}S_{j}L_{j}$$

In which S_j_ is the slowness and L_j_ is the length of a ray-path which can be computed directly from the distance between the two points. As an illustration, the travel times for node “a” in Figure 3 are stored as characteristic input data alongside the direction wave paths. The smallest travel time amongst the other paths in Figure 3, is counted as the characteristic travel time from node (a) to node (b). By using the SIRT technique the inversion process is performed in which the difference between theoretical and observed travel time to the cells intercepted by the ray is distributed. The length of the ray path is taken into account as a variable to make the distribution.

(2) $$S_{i}=\frac{\sum_{j}\Delta_{S_{\text{ij}}}L_{\text{ij}}}{\sum_{j}L_{\text{ij}}}$$

(3) $$∆S_{\mathrm{ij}}=\frac{\left(\sum_{j}{\Delta T}_{i}L_{\text{ij}}\right)}{{\left(\sum_{j}L_{\text{ij}}\right)}^{2}}$$

in which ∆T_i_ is the difference in travel time between the theoretical and observed values. The inversion process is repeated to amend the distributed value until the total slowness of the ray finally falls into an acceptable range of discrepancy with the observed data.

### 2.2. General Approach for Verifications of Proposed Algorithms

**Figure 4:**
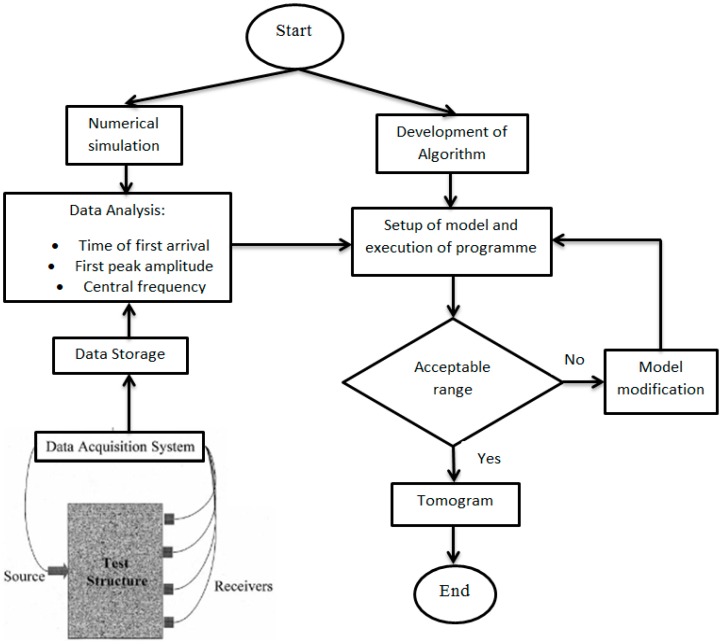
Summary of study methodology.

Three types of tomography reconstruction algorithms were developed based on stress wave parameters. The developed algorithms were verified with various numerical simulation models and experimental specimens. The developed algorithms of tomography were used to detect and visualize defects in the form of honeycomb and partially grouted tendon sheath in concrete block specimens. Numerical and experimental results are presented and discussed in the following sections. The summary of the study methodology is displayed in Figure 4.

## 3. Numerical Simulation and Case Studies

To generate wave data for verifying the developed algorithms, two-dimensional (2D) numerical simulations of wave motions were conducted with commercially available software (Wave 2000) [22]. The simulated data were processed to be used as input data for tomography reconstruction. The fundamental equation of two-dimensional propagation of elastic waves in an elastic medium is given as follows:

(4) $$p\frac{\partial^{2}u}{\partial t^{2}}=\mu \nabla^{2}u+\left(\lambda +\;\mu \right)u$$

where u = u(x,y,t) is the time-varying displacement vector, p is the material density, λ and μ are the first and second lame constants, and t is the travel time. The software performs computation to solve Equation (4) at discrete points with respect to the boundary conditions of the model, which include the input source that has been defined as the time-dependent displacement at a given location under a set of initial conditions. The above equation is applicable for solving wave propagation in any heterogeneous geometry, while the continuity conditions for stress and strains must be satisfied on the interfaces. The wave speed of simulation can be determined by parameters such as material density p, the first and second lame constants, λ and μ. The longitudinal wave speed, V_L_ in the medium is given by [23]:

(5) $$V_{L}=\sqrt{\frac{\lambda +2\mu }{p}}$$

The relationship between Young’s modulus of medium with the first and second lame constants, λ and μ is given by

(6) $$E=\frac{\mu \left(3\lambda +2\mu \right)}{\lambda +\mu }$$

**Figure 5:**
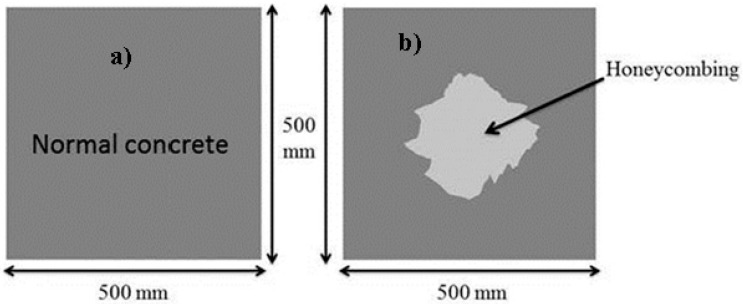
Simulation models showing (**a**) normal concrete structure and (**b**) concrete structure with honeycomb.

A 500 mm square concrete medium was considered for wave propagation simulation purposes as presented in Figure 5. The first defect in concrete was defined in the form of honeycomb. The essential material properties required for simulation are presented in Table 1.

**Figure 6:**
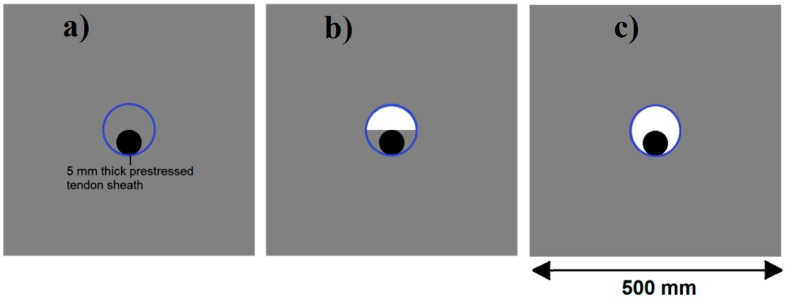
Schematic diagrams of simulated pre-stressed concrete with steel tendon models showing (**a**) 100%; (**b**) 50% filling grout tendon and (**c**) 0% grout filling inside the tendon duct.

The next simulation model resembled that of a pre-stressed concrete medium with a tendon duct and circular steel tendon as reinforcement, as shown in Figure 6. The concrete was modelled to be 500 mm square. The diameter of the tendon duct, thickness of tendon sheath wall, and the diameter of the steel tendon at the center of the tendon duct were modelled as 100 mm, 5 mm, and 50 mm, respectively. The grout filling inside the tendon duct was varied at 100%, 50%, and 0% (ungrouted) in separate simulations.

**Figure 7:**
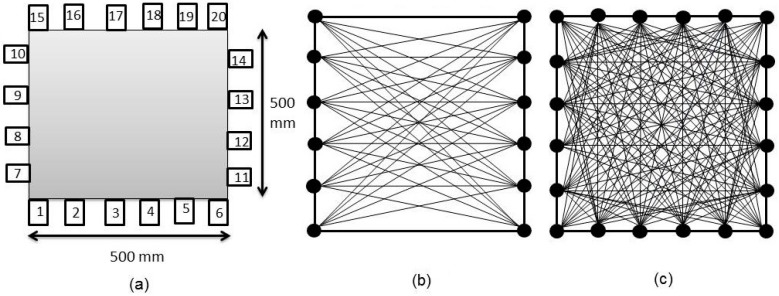
(**a**) Arrangement of sensors on 500 mm × 500 mm concrete model; (**b**) ray-path coverage with 2-side sensor array; (**c**) ray-path coverage with 4-side sensor array (complete coverage).

The numerical simulation process was performed with two different sets of sensor array: two-sided placement (left and right sides of model) and full coverage (four-sided sensor placement). Figure 7 shows the schematic diagrams of the sensor arrangement and imaginary wave network. The spacing between two adjacent sensors was 88 mm. One of the sensors acts as the trigger sensor for a source impact located near to it. The simulated source was configured as excitations by impact (point source) which was a single cycle of Sine Gaussian pulse at 50 kHz frequency. All other sensors mounted on a different side against the trigger were be set as receivers to record the transmitted wave. The recorded wave data was then further analyzed for travel time and attenuation properties computation. In tomography reconstruction, models consisting of 25 and 100 square elements were employed.

## 4. Simulated Wave Motions

**Figure 8:**
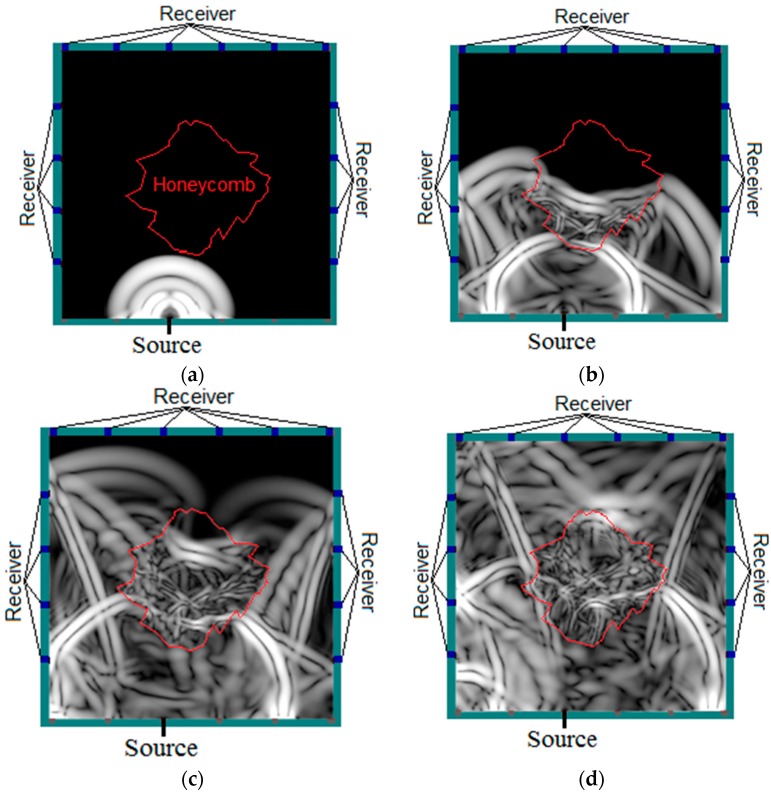
Four consecutive snapshots of the simulated transient displacement field in the model with honeycomb: (**a**) Wave starts to propagate from a trigger; (**b**) Wave experiences distortion by honeycomb; (**c**) Wave propagated faster in normal medium than in honeycomb; (**d**) Wave-front arriving at the side and being received.

Figure 8 displays the four consecutive snapshots of simulated elastic wave propagation through the concrete model with honeycomb. It could be observed that as the wave propagated from intact regions and encountered honeycomb regions, refraction, reflection and scattering of the wave took place. As a result, the energy of the wave was distorted and it propagated at a lower speed compared to propagating in homogeneous concrete as indicated in Figure 8b. The delay in wave travel time became more visible as it propagated further into the honeycomb, with reflection and scattering being observed at the honeycomb-concrete interface due to the acoustic impedance difference, as seen in Figure 8c. After a while, the wave arrived at the sides of the model being received by all sensors, as shown in Figure 8d.

**Figure 9:**
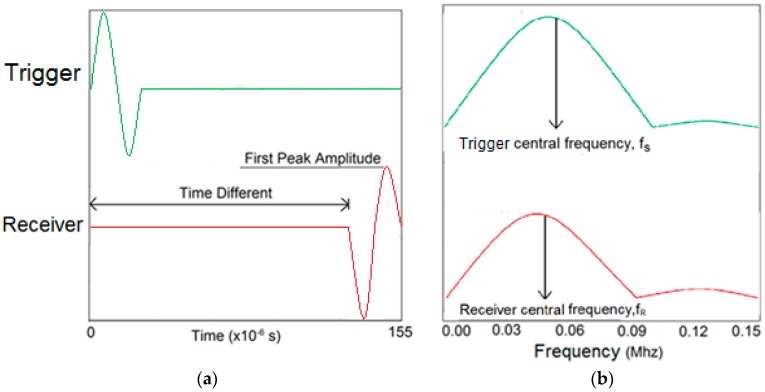
Examples of elastic wave time-domain (**a**) and their spectral amplitude (**b**) for sensor at a trigger and a receiver.

Figure 9a is an example of time domain data for a trigger and a receiver as simulated. The time difference between excitation from trigger and wave arrival at the receiver is distinguishable. The time domain data was converted to frequency domain by fast Fourier transform (FFT), yielding the amplitude spectrum of the waves as shown in Figure 9b. The centroid frequency and variance could be computed from the amplitude spectrum.

It can be noticed that the central frequency at the receiver has been shifted to the left side (lower frequency) which is an implication of the occurrence of attenuation in the higher frequency components as a result of wave distortion by the honeycomb.

## 5. Tomograms

Tomograms were plotted on the basis of distributions of pulse velocity, amplitude, and frequency attenuation coefficient, and medium quality factors. For all the aforementioned types of tomograms, signal properties are commonly defined by the first detectable disturbance of the received waveform. The specific conditions of defects and size of meshing along with the corresponding tomograms are discussed in the following.

### 5.1. Travel Time Tomography

Pulse velocity for a specific wave path (from the trigger to receiver) can be computed by dividing the wave path length by its travel time. The theoretical travel time can be computed by using the ray-trace technique with assumption of uniform wave velocity in individual cells on the mesh. The ray-path is formed as connected segments that are described as lines between arbitrary combinations of two nodal points. On the basis of the assumptions, the travel time for a wave to propagate from trigger sensor to destination sensor is as follow:

(7) $$t=\int_{A}^{B}1/v\;\mathrm{dl}=\int_{A}^{B}s\;\mathrm{dl}$$

where, v is velocity, s is the wave slowness (also known as reciprocal of velocity), and dl is the length of the ray path.

**Figure 10:**
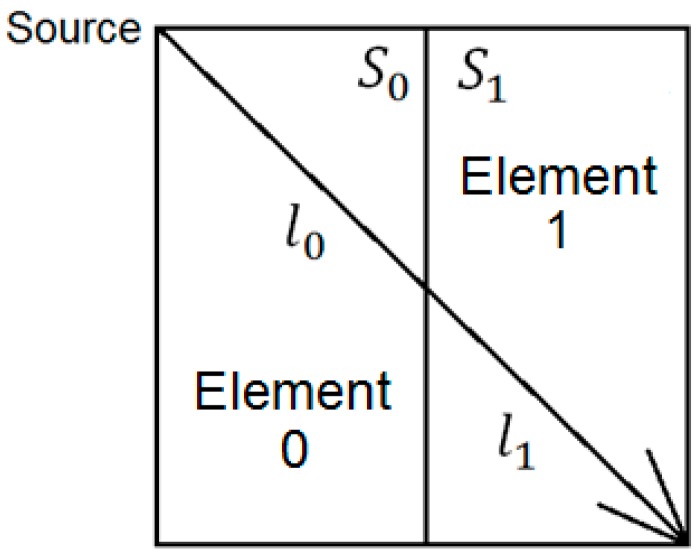
Illustration of a wave ray propagating in two elements from top left to bottom right.

A square area is divided into two elements as shown in Figure 10. Given that the length of a ray triggered from a source and propagating inside element “0” is l_0_, its slowness is designated as S_0_. The difference between the observed travel time and expected travel time for source “0” is as follow:

∆T = T_observed_ − T_theory_ (8)

and the difference of slowness in element “0” is as follow:

(9) $$∆S_{0}=\frac{∆T_{0}}{L}$$

In which L is the total length of the ray from trigger point to receiver point (node) in the object. The difference has to be updated during the iterative computation process.

On occasions where there is more than one ray passing through the same element, Equation (3) can be modified to address the change of slowness at element j as follow:

(10) $$∆S_{j}=\frac{\sum^{\text{​}}\frac{∆T_{i}}{L_{i}}{\text{x l}}_{j}}{\sum^{\text{​}}l_{j}}$$

where l_j_ represents the length of ray at element j, i represents the ray path number and j represents the element number. Therefore, the updated slowness for element j, S_j_ is as follow:

(11) $${S_{j}}_{\mathrm{updated}}={S_{j}}_{\mathrm{inputted}}+∆S_{j}$$

By making use of the above principle, different travel time tomography (TTT) cases were examined. The first case investigates a square cross section of 500 mm of concrete with a honeycomb in the center, as depicted in Figure 5. The medium is considered to be a normal concrete (P-wave velocity, C_p_ = 4000 m/s) and the honeycomb void is air (C_p_ = 300 m/s). The excitation was driven consequently to 12 and 20 different positions on two and four sides, respectively, and each time the sensors at the opposite side acted as receivers. In general from Table 2, it can be conferred that reduction of the velocity illustrated in the travel time tomography is associated with the inhomogeneity void existing in the honeycomb placed in the medium. It is reasonable that when the honeycomb is positioned on the straight line between the excitation and receiver location, the travel time becomes longer due to the lack of the presence of the shortest path (straight) for the wave to propagate. Comparing Figure 8a,b shows that when P-wave impinges on the boundary of the honeycomb, the wave-front is no longer uniform and the wave propagates on the surface of the honeycomb. This causes a delay in travel time from trigger point to receiver location. Therefore, an additional amount of traveling time from trigger point to receiver results in lower measured velocity for the paths including the honeycomb. It is obvious that the four-sided measurement provides better visualization and resolution with higher level of precision due to the increase of input data for the reconstruction process compared to the two-sided tomography. On the other hand, travel time tomography showed lesser sensitivity to the meshing size since the resolution for the 100 elements case was slightly better than the 25 elements case.

**Figure 11:**
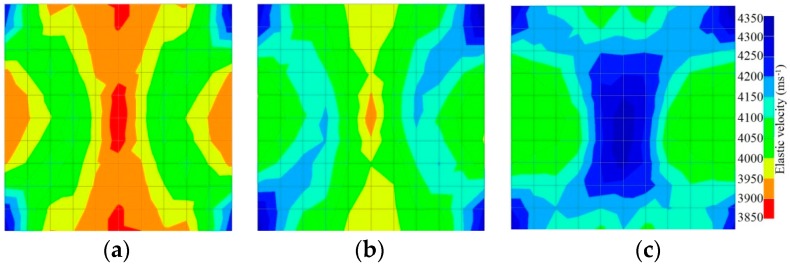
Travel time tomography results of honeycombed concrete model based on measurements using a 2-sided sensor arrangement: (**a**) 0%; (**b**) 50%; and (**c**) 100% grout filling.

**Figure 12:**
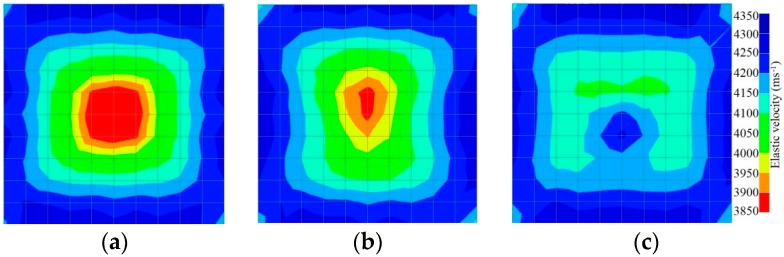
Travel time tomography results of of honeycomed concrete model based on measurements using a 2-sided sensor arrangement: (**a**) 0%; (**b**) 50%; and (**c**) 100% grout filling.

Figure 11 and Figure 12 show tomography results of the PC model with 2-sided and 4-sided sensor attachments, respectively. It can be confirmed that the visualization to spot the 0% and 50% grout filling conditions was improved using a 4-sided sensor arrangement as shown in Figure 12a,b. For 100% grout filling, both the center of the 2- and 4-sided sensor attachments of the travel time technique showed a center with blue color as in Figure 11c and Figure 12c which indicates the efficiency of 100% filling of the duct with the grout can be visualized properly.

### 5.2. Amplitude Tomography

The attenuation of elastic waves is represented by the decrease in wave amplitude due to reduced propagation energy. This decline in energy is usually caused by both the changes in intrinsic properties of the material and geometrical spreading [24]. Subsequently, this phenomenon provides a way to extract medium property information by analyzing the decay of the elastic wave amplitude. The loss of wave energy is mostly attributed to scattering and absorption phenomena. Scattering attenuation is caused by heterogeneity in the material such as the existence of different acoustic impedance property in a medium or the presence of fractures [25]. On the other hand, intrinsic attenuation or absorption is because of internal friction of the material. Attenuation of elastic waves is highly governed by the inelastic properties of the medium, while the travel time of the elastic wave depends on the elastic properties and medium density [26,27,28,29,30].

To deal with amplitude attenuation, it is necessary to define the wave peak amplitude. Theoretically, the first arrival wave peak amplitude at the receiving point can be defined as

(12) $$A_{i}=A_{o}e^{-\alpha L}$$

where, $A_{o}$ is the source amplitude, α is the attenuation coefficient of the medium. The coefficient, α is used to indicate the penetration of the wave and its speed when propagating in a medium. The existence of a void and defect can be recognized when the arriving wave energy is either irregular or distorted. In the proposed algorithm, the first receiving offset amplitude from the source wave propagations is computed from Equation (12). It is presumed that the presence of a void or defect inside the medium results in a change in the attenuation coefficient from which an updated amplitude can be computed. Thus, to update amplitude changes, modification to Equation (6) has to be taken into account as follow:

(13) $$A_{r}=A_{o}e^{-\left(\alpha +\;∆\alpha \right)L}$$

With reference to a typical two element model shown in Figure 10, the change of attenuation coefficient for element “0” can be calculated as

(14) $$∆\alpha_{0}=\frac{\left[\;\ln\left(\frac{A_{r}}{A_{o}}\right)\;+\;\sum^{\text{​}}\alpha l\right]}{L}$$

in which l represents the ray path of the ray in element “0”. For occasions where there is more than one ray passing through that element, Equation (14) has to be modified to address the change of attenuation coefficient at element number j as below

(15) $$∆\alpha_{j}=\frac{\sum^{\text{​}}\left(\frac{-\ln{\left(\frac{A_{r}}{A_{o}}\right)}_{i}-\sum^{\text{​}}{\alpha l}_{i}}{L_{i}}\right)\;\times {\;l}_{\text{ij}}}{\sum^{\text{​}}l_{\text{ij}}}$$

Therefore, the updated α for element “j” can be expressed as

α_j_ (updated) = α_j_ (inputted) + ∆α_j_ (16)

By making use of the above principle, different amplitude tomography cases were examined. The first case investigates a square cross section of 500 mm of concrete with a honeycomb in the center, as depicted in Figure 5. It is reasonable that when the honeycomb is positioned on the straight line between the excitation and receiver location, the amplitude drops due to the presence of a defect which is the result of diffraction and scattering of the waves. Therefore, the attenuation coefficient becomes higher when elastic waves encounter honeycomb (Table 3). The tomograms with 25 elements display larger honeycomb than tomograms plotted with 100 elements. This might be attributed to the nature of the amplitude tomogram, for which the decrease of amplitude is due to scattering and absorption of wave energy by the honeycomb. Therefore, tomograms with 100 elements provide sufficient meshing to compute the amount of scattering and eliminate reflection from the iteration process, whereas tomograms with 25 elements are not able to refine the iteration process.

In addition, the surroundings of the tomogram plotted by techniques show a higher attenuation value. This is because of the wave reflection when it arrives at the side and adjacent to the receiver sensor. The reflected wave is normally weak in elastic energy with low amplitude compared to the original wave front.

**Figure 13:**
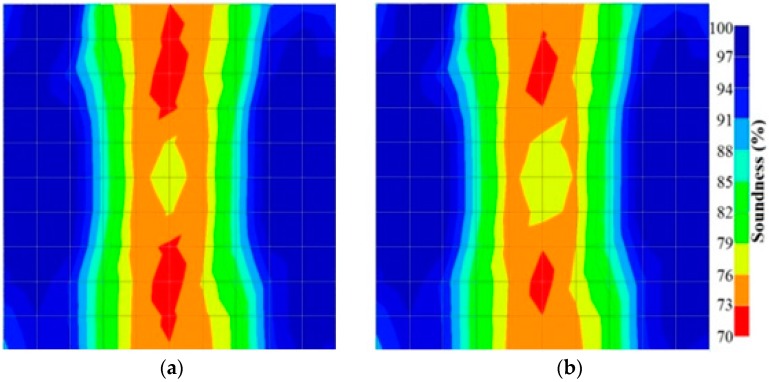
Soundness of grout filling in the concrete model based on amplitude tomography results of the 2-sided sensor arrangement: (**a**) 50% and (**b**) 0% fillings.

**Figure 14:**
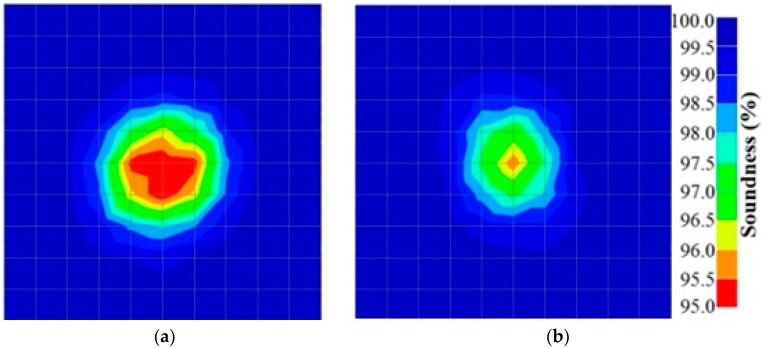
Soundness of grout filling in the concrete model based on amplitude tomography results of the 4-sided sensor arrangement: (**a**) 0% and (**b**) 50% fillings.

Figure 13 and Figure 14 give visualizations of the PC model computed using the amplitude tomography algorithm. Based on the results, it was found that the 2-sided sensor arrangement was less capable of spotting precisely the presence of void in 0% and 50% grout filling cases as shown in Figure 13. However, the amplitude tomogram by the 4-sided sensor arrangement could fairly illustrate the void for the case of 0% filling.

### 5.3. Frequency Tomography

The frequency tomography is based on computing the frequency variation of waves as they propagate in a medium from a source to a destination. Quan and Harris [31] proposed that the product of multiplying the attenuation coefficient wave by the length of the ray-path in a material can be expressed as

(17) $${\int^{\text{​}}}_{\text{ray}}{\;\alpha }_{0\;}\text{dl}=\left(f_{S}-f_{R}\right)/\sigma_{S}^{2}$$

in which α_0_ is intrinsic attenuation coefficient, f_s_ is the centroid frequency of input data, f_R_ is the centroid frequency of received data and σ_s_^2^ is the variance. For the case of variance which is a constant, the change of attenuation coefficient with intrinsic attenuation coefficient, $\alpha_{o}+∆\alpha$ can be estimated based on the centroid downshift of frequencies of the input spectrum and the output spectrum. Thus, In the case of the existence of void or defect, differences in the attenuation coefficient value would occur resulting in the following equation:

(18) $$\left(\alpha_{0}+∆\alpha \right)l=\frac{\left(f_{S}-f_{R}\right)}{\sigma_{s}^{2}}$$

By assuming the typical model as shown in Figure 10, the change imposed to the attenuation coefficient for element 0 is calculated as follows:

(19) $$∆\alpha_{0}=\frac{\left[\frac{\left(f_{S}-f_{R}\right)}{\sigma_{s}^{2}}-\;\sum^{\text{​}}\alpha l\right]}{L}$$

In events where there is more than one ray passing through that element, Equation (16) has to be modified to address the change of attenuation coefficient at element j as below:

(20) $$∆\alpha_{j}=\frac{\sum^{\text{​}}\left(\frac{-{\frac{\left(f_{S}-f_{R}\right)}{\sigma_{s}^{2}}}_{i}-\sum^{\text{​}}{\alpha l}_{i}}{L_{i}}\right)\;\times {\;l}_{\text{ij}}}{\sum^{\text{​}}l_{\text{ij}}}$$

Therefore, the updating of the attenuation coefficient for element “j” is calculated by using Equation (16).

By making use of the above principle, different frequency tomography cases were examined. The first case investigates a square cross section of 500 mm of concrete with a honeycomb in the center, as depicted in Figure 5. It can be observed that when the honeycomb is positioned on the straight line between the excitation and receiver location, the frequency drops due to the presence of a defect which is the result of diffraction and scattering waves. Therefore, the attenuation coefficient becomes higher when elastic waves encounter the honeycomb (Table 4).

The elastic wave frequency dropped due to the presence of honeycomb and could be justified by a fundamental concept of physics known as the pendulum principle [1]. It is given that the frequency of pendulum f and its length l are in inverse relation:

f^−1^ = 2π(l/g)^1/2^ (21)

When there was an increase in the crack size, either attributed to the enlargement of one previous crack or the coalescence of several previous smaller cracks, or both, the AE signal correspondingly turned out to be of progressively lower frequency, until leaving the ultrasonic range and attaining the sonic range it was identified by the well-known seismic roar. Again it can be observed that the tomogram computed from a 4-sided measurement exhibited precise indication of the honeycomb.

**Figure 15:**
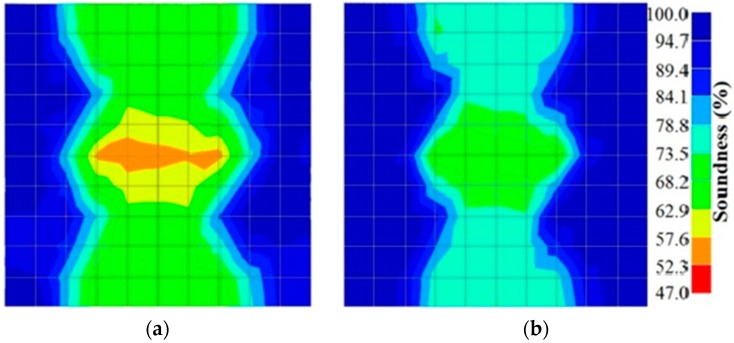
Soundness of grout filling in the concrete model based on frequency tomography results of the 2-sided sensor arrangement: (**a**) 0% and (**b**) 50% fillings.

**Figure 16:**
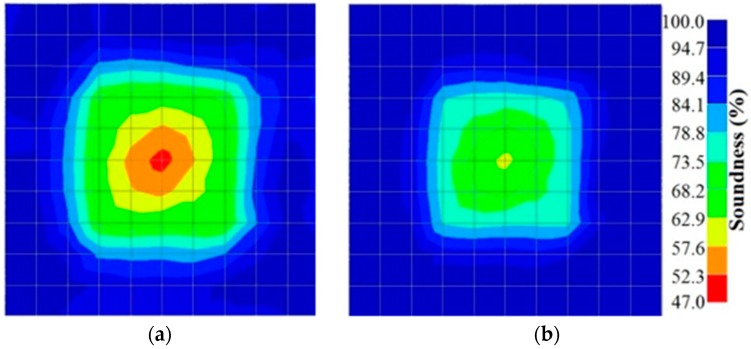
Soundness of grout filling in the concrete model based on frequency tomography results of the 4-sided sensor arrangement: (**a**) 0% and (**b**) 50% fillings.

However, in the second case, frequency tomograms for the PC model did not provide indicative results except for the case of the tomogram for a 4-sided measurement of the 0% grout filling. However the void illustrated by this tomogram seems to be smaller than the defined duct in the 0% grout filling case. The soundness results are presented in Figure 15 and Figure 16.

### 5.4. Medium Quality Factor (Q-Value) Approach

By using analyses of wave attenuation and offset time of the P-wave an alternative approach to estimate the properties of the medium is proposed. Medium quality factor, Q is a seismic characteristic of a medium’s attenuation property and is useful for improving tomogram resolution [30]. By using the Q-value, the quality of the medium can be determined and the tomogram of the medium can be reconstructed. The medium’s quality factor provides an alternative to data to plot the tomogram. The medium quality factor, Q-value can be revised as

(22) $$Q=\;\frac{\pi }{\alpha v}$$

where values for the attenuation coefficient, α and the elastic velocity v of the element were obtained from the travel time tomography and amplitude tomography techniques. Therefore, the assessment process can be extended to obtain the Q-value for each element of the testing medium.

By using the above concept, the Q-value tomogram is obtained. The first case under investigation was the concrete model with honeycomb, from which the Q-value tomography results are presented in Table 5. Again it can be observed that the Q-value tomograms with 100 elements could indicate the presence of good honeycomb resolution, especially in the cases where soundness was used as a parameter. In addition, contrary to the amplitude and frequency tomography, the accuracy of Q-value tomography is considered independently of the number of elements and mesh size because it is not affected by the energy of excitation.

**Figure 17:**
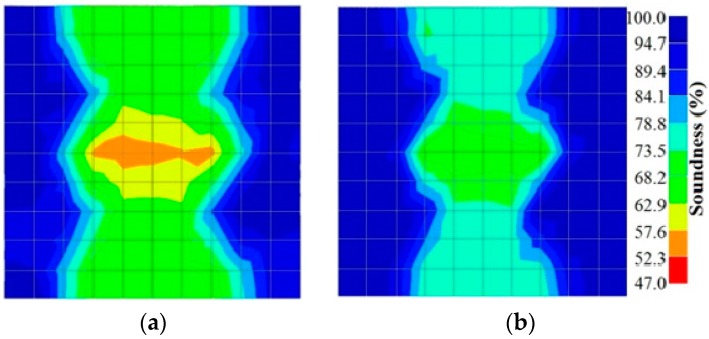
Soundness of grout filling in concrete model based on Q-value tomography results of the 2-sided sensor arrangement: (**a**) 0% and (**b**) 50% fillings.

**Figure 18:**
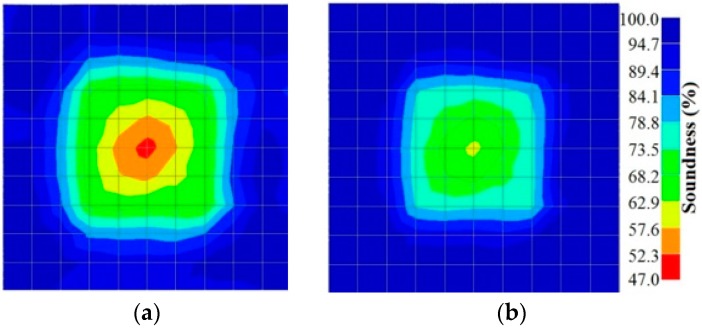
Soundness of grout filling in the concrete model based on Q-value tomography results of the 2-sided sensor arrangement: (**a**) 0% and (**b**) 50% fillings

Figure 17 and Figure 18 show the results of the Q-value tomography concrete model with tendon duct. The 0% and 50% grout filling conditions were indicative of reconstructions using 100 elements.

## 6. Experimental Case Study

**Figure 19:**
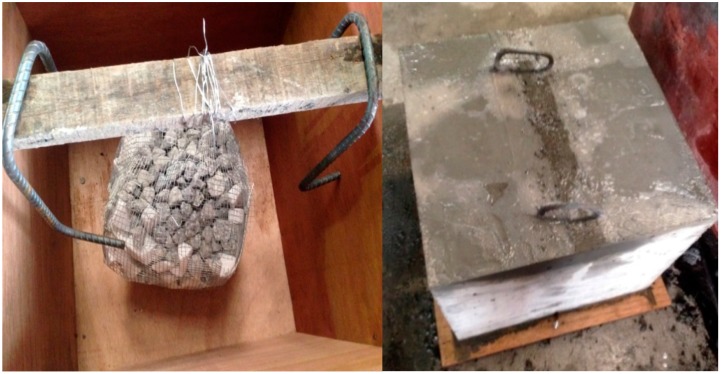
Concrete sample with a honeycomb placed at the center.

To verify the numerical simulation data, two concrete samples were prepared to be measured using the developed tomography techniques. The first one was a 500 mm concrete cubic sample in which a honeycomb was intentionally “modelled” at the center as shown in Figure 19. The honeycomb was made by wrapping up crushed granite stones with a steel wire net to form a ball like object that had a maximum diameter of 200 mm and a density of 900 kg/m^3^. The concrete for the sample was tested as having a 28-day compressive strength of 55 MPa.

The instrumentation setup consisted of single crystal material piezoelectric accelerometers and a computer-based data acquisition device (NI PXle-1073). The sampling rate was set at 20 kHz. The duration of the recording of the waveform after excitation was set at 0.01 s in order to generate a stress wave that could be detected by a piezoelectric transducer. In this experiment, steel spheres of 3 mm and 19 mm were used to produce stress waves with frequency of 25 and 10 kN.

**Figure 20:**
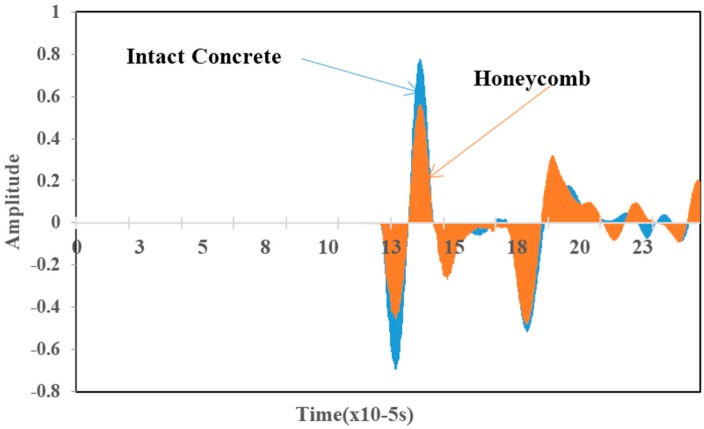
Amplitude *vs.* time data of measured waves.

**Figure 21:**
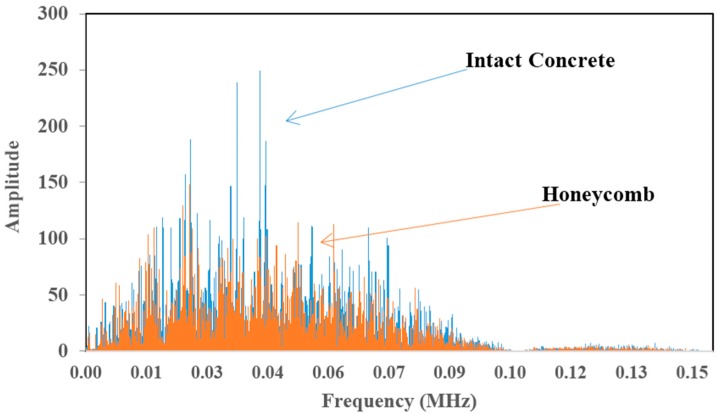
Amplitude *vs.* frequency.

The results pertaining to the ultrasound signals crossing the honeycomb are presented in Figure 8, Figure 20 and Figure 21. Figure 8a shows the wave propagation through the concrete which contains a honeycomb in the center of the medium. Figure 8b shows the moment when waves come close to the honeycomb and propagate through it with slower velocity compared to the intact concrete medium. However, it is obvious that the waves are able to pass through the whole concrete medium, including those areas with honeycomb as in Figure 8c,d.

Figure 20 is an example of time domain wave data of amplitude *vs.* time for waves that propagates in two types of medium: intact concrete and concrete with honeycomb. Figure 20 shows that the amplitude of the wave propagated in concrete with honeycomb is lower than in intact concrete. Besides, Figure 20 demonstrates that waves in intact concrete reached the sensor earlier than waves that were propagated in concrete with honeycomb.

Figure 21 is a graph of amplitude *vs.* frequency for a wave that propagates in two types of medium: intact concrete and concrete with honeycomb. Figure 21 illustrates that the graph is shifted to the right for intact concrete compared to concrete with honeycomb. In Figure 21, the standard deviation of the wave propagated in sound concrete was recorded as 30.02 for amplitude and 0.052 MHz for frequency measurements while for concrete with honeycomb, the registered values were 22.93 and 0.048 MHz for amplitude and frequency, respectively. Thus, the results show that the wave experienced reflection in concrete with honeycomb and some part of the energy was transformed to another form so that the amplitude and frequency that the sensor received for concrete with honeycomb were less than for intact concrete.

**Figure 22:**
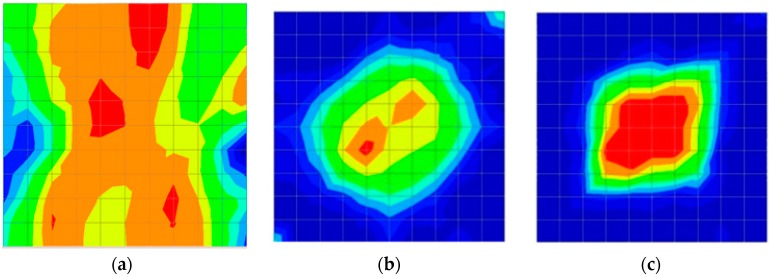
Soundness of the tomograms of honeycomb: (**a**) 2-sided velocity; (**b**) 4-sided amplitude; (**c**) 4 sided frequency.

The results for travel time (velocity), amplitude, and frequency tomograms are presented in Figure 22. It can be observed that the efficacy of the presented algorithm was demonstrated as able to provide the appropriate resolution of artificial honeycomb in concrete.

**Figure 23:**
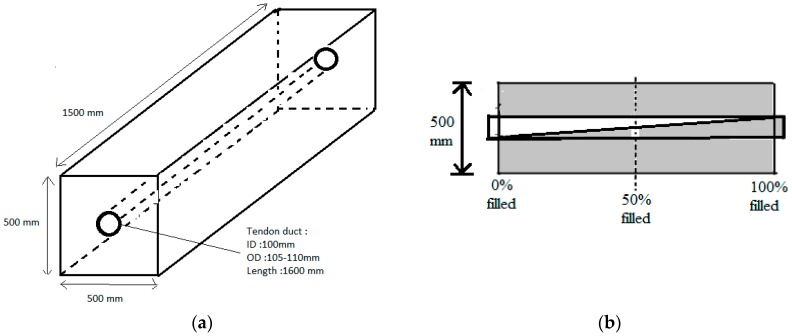
(**a**) 3D view of pre-stressed concrete (PC); (**b**) Left hand side of PC specimen that had been cast.

The next case study was a PC specimen which was prepared based on a design illustrated in Figure 23. Ultrasonic tomography was tested to detect the artificial defect in the cross section caused by a different percentage of void in the PC duct. This void was placed by using different percentages of cement grout as duct filler. The three grout percentages were 100%, 50%, and 0% which are in compliance with the simulated PC conditions in the numerical wave analysis. The pre-stressed tendon duct was a 1600 mm steel duct (ID: 100 mm, OD: 107 mm). The grout mix was designed in compliance with the ASTM C 476 requirement. The compressive strength of concrete was 55 MPa at 28 days.

However, the instrumentation setup generally consists of piezoelectric sensors and a computer-based data acquisition device. In order to generate a stress wave that can be detected by a piezoelectric transducer, a small steel sphere was used to tap against the surface of the testing concrete. A small steel sphere typically ranges from 3 mm to 15 mm in diameter.

**Figure 24:**
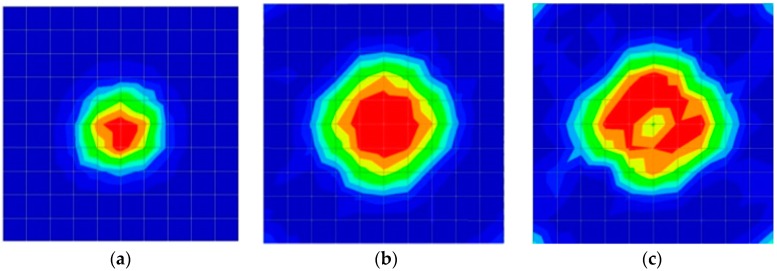
Soundness of tomograms for duct of PC with 0% filling: (**a**) 2-sided velocity; (**b**) 4-sided amplitude; (**c**) 4 sided frequency.

The results for travel time, amplitude, and frequency tomograms are presented in Figure 24. It can be observed that the efficacy of the presented algorithm was demonstrated as being able to provide an appropriate resolution of artificial honeycomb in concrete. However, the result of the velocity tomogram was more precise.

## 7. Discussion

Utilizing the above configuration it became possible to develop an algorithm to plot tomograms for some indicative elastic wave parameters in the case of inhomogeneous materials. At this point it seems to be necessary to discuss the significant aspects of the whole procedure. The general procedure seems to be considerably sensitive to the existence of a deteriorated material zone such as honeycomb rather than to a central void like PC duct.

In addition, the level of sensitivity to the inhomogeneity size that can be determined is profoundly dependent on the sensor placement, as well as the tomography element size, particularly, for amplitude, frequency, and Q-value tomograms. In the present study, the distance between neighboring sensors was 88 mm, while the tomography cell sizes were 100 mm and 50 mm for tomograms with 25 and 100 elements, respectively. This can be the reason why there was no high resolution tomography for some tomograms with 25 elements; except in cases where the large dimension of the vertical deterioration zone (honeycomb) was much larger than the cell size.

It is noteworthy that in the experimental procedure the excitation frequency is one of the significant parameters. The excitation frequencies concern the wavelength/defect size ratio (λ/d). The applied frequency can result in a higher and lower ratio than unity which is in direct relation to the tomograms’ resolutions and cell size.

The above aforementioned results imply that without changing the raw information (number of wave paths examined or number of sensors placed in the field) the results can be enhanced by selecting a smaller tomography mesh size. The results suggest that the cell could be set to a value smaller than the distance between adjacent sensors. However, the smallest improvement in tomogram resolution is helpful, since the experimental procedure is in any case the most problematic task in the monitoring of a large structure. Although, a required time for computation on the tomography software is required to conduct the necessary iterations, it is minor compared to a better understanding of the internal situation of a structure, while it is negligible considering contemporary computational tools.

## 8. Conclusions

In this study, the application of different parameters of elastic stress waves by numerical and experimental analysis was investigated. The EWT technique for different types of defects was investigated by developing reconstruction procedures utilizing different parameters of elastic waves, namely the travel time, wave amplitude, wave frequency, and Q-value. The following conclusions were made from the present study.

- Ray tracing of the travel time tomography technique has to be introduced into the amplitude and frequency tomography technique in order to obtain the ray-path of the wave using the ray-tracing method. This is because the elastic wave experiences refraction and reflection and thus does not necessarily propagate only in a straight line.
- The amplitude of the elastic wave propagation in concrete undergoes a much greater change because of scattering and absorption compared to the delay in travel time due to inhomogeneity of the medium. This phenomenon indicates the high sensitivity of elastic wave attenuation for potential adoption in the evaluation of soundness in concrete.
- The surroundings of amplitude, frequency, and Q-value tomograms might indicate low soundness because of lack of ray information and receiver sensors that record the reflected waves that arrive at the adjacent.
- Travel time tomography is less sensitive among four types of tomography techniques. Thus, it is recommended to conduct other tomography techniques to compare, interpret, and confirm the experimental results.
- By reducing the element size or increasing the number of elements, the visualization of a tomogram could be improved significantly.

## Figures and Tables

**Table 1:** Material properties of testing medium.

Material	Density, p (kg·m^−3^)	Young‘s Modulus, E_c_ (GPa)	First Lame Constants, λ (GPa)	Second Lame Constants, μ (GPa)	Longitudinal Wave Speed, V_L_ (m·s^−1^)
Concrete	2400	35.71	12.4	13	4000
Honeycomb	1600	12.78	1.6	1	1500
Aluminium	2700	69	61.38	24.95	6420
Polyethylene	920	2	2.962	0.2683	1950
Steel	7830	200	121.2	80.77	6001

**Table 2:** Travel time tomography reconstruction results for concrete honeycomb detection based on simulated wave data.

Description	Sound Concrete	Honeycombed Concrete	Soundness
2-sided with 25 elements	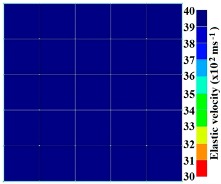	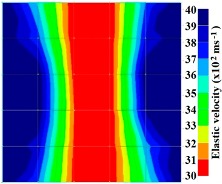	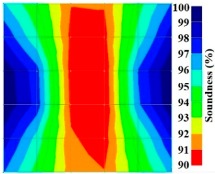
4-sided with 25 elements	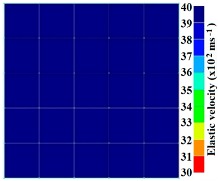	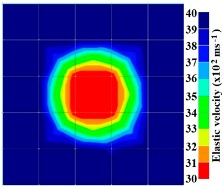	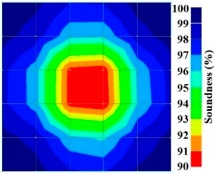
2-sided with 100 elements	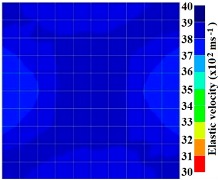	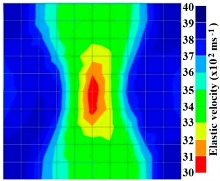	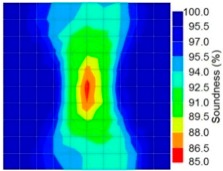
4-sided with 100 elements	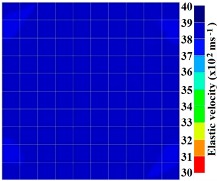	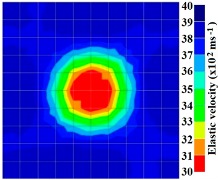	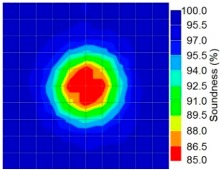

**Table 3:** Amplitude tomography reconstruction results for concrete honeycomb detection based on simulated wave data.

Description	Sound Concrete	Honeycombed Concrete	Soundness
2-sided 25 elements	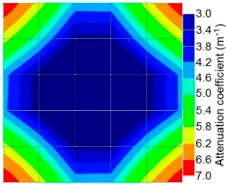	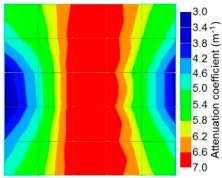	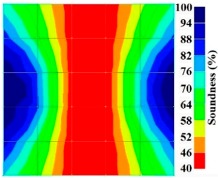
4-sided 25 elements	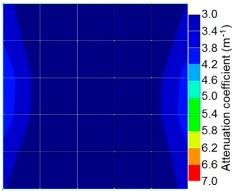	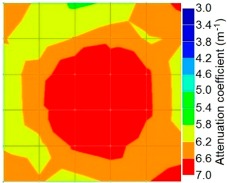	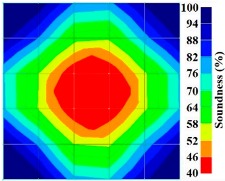
2-sided 100 elements	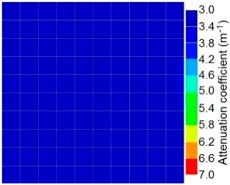	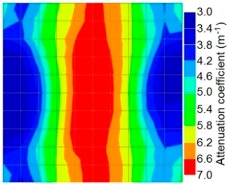	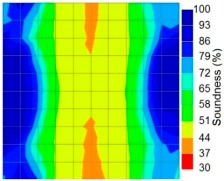
4-sided 100 elements	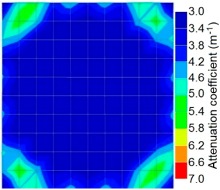	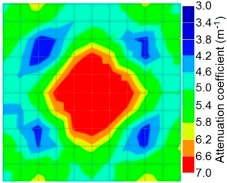	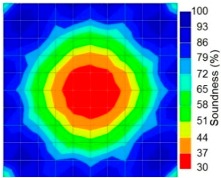

**Table 4:** Frequency tomography reconstruction results for concrete honeycomb detection based on simulated wave data.

Description	Sound Concrete	Honeycombed Concrete	Soundness
2-sided	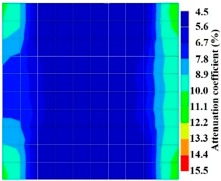	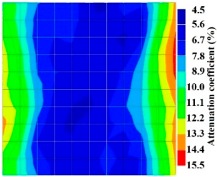	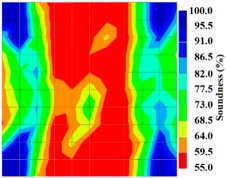
4-sided	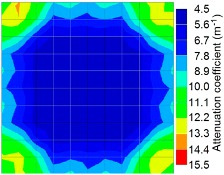	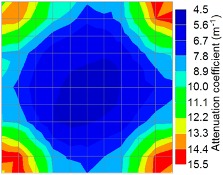	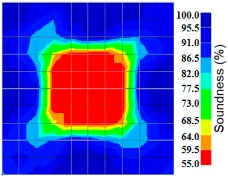

**Table 5:** Q-value tomography reconstruction results for the concrete model with honeycomb.

Description	Sound Concrete	Honeycombed	Soundness
2-sided with 100 element	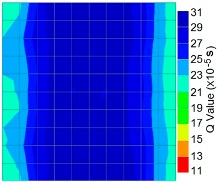	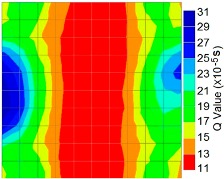	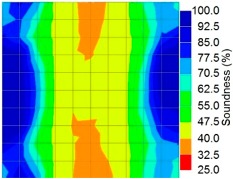
4-sided with 100 element	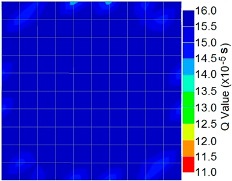	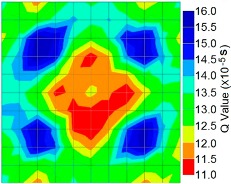	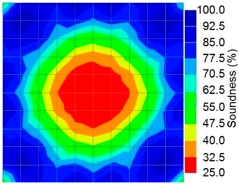
4-sided with 100 elements